# Estimating and mitigating amplification bias in qualitative and quantitative arthropod metabarcoding

**DOI:** 10.1038/s41598-017-17333-x

**Published:** 2017-12-15

**Authors:** Henrik Krehenwinkel, Madeline Wolf, Jun Ying Lim, Andrew J. Rominger, Warren B. Simison, Rosemary G. Gillespie

**Affiliations:** 1 Department of Environmental Sciences, Policy and Management University of California Berkeley Mulford Hall, Berkeley, California USA; 2 Center for Comparative Genomics California Academy of Sciences Music Concourse Drive, San Francisco, California USA

## Abstract

Amplicon based metabarcoding promises rapid and cost-efficient analyses of species composition. However, it is disputed whether abundance estimates can be derived from metabarcoding due to taxon specific PCR amplification biases. PCR-free approaches have been suggested to mitigate this problem, but come with considerable increases in workload and cost. Here, we analyze multilocus datasets of diverse arthropod communities, to evaluate whether amplification bias can be countered by (**1**) targeting loci with highly degenerate primers or conserved priming sites, (**2**) increasing PCR template concentration, (**3**) reducing PCR cycle number or (**4**) avoiding locus specific amplification by directly sequencing genomic DNA. Amplification bias is reduced considerably by degenerate primers or targeting amplicons with conserved priming sites. Surprisingly, a reduction of PCR cycles did not have a strong effect on amplification bias. The association of taxon abundance and read count was actually less predictable with fewer cycles. Even a complete exclusion of locus specific amplification did not exclude bias. Copy number variation of the target loci may be another explanation for read abundance differences between taxa, which would affect amplicon based and PCR free methods alike. As read abundance biases are taxon specific and predictable, the application of correction factors allows abundance estimates.

## Introduction

Next generation sequencing technology has ushered in a revolution in evolutionary biology and ecology, enabling analyses at unprecedented throughput and detail^1^. This revolution has spurred various studies in the field of metabarcoding. Next generation sequencing-based metabarcoding comes with a small workload, is cost efficient^2^, and provides ecologists with a means to identify large numbers of taxa in a given community. The resulting leap in throughput has allowed large-scale metabarcoding of entire ecosystems^3–6^ and promises unprecedented insights into ecosystem function and assembly through the recovery of species richness, food web structure, cryptic species, and hidden diversity, such as internal parasitoids^7–10^. Nevertheless, a critical, but not yet sufficiently understood, application of metabarcoding approaches is the potential estimation of species abundances^11^.

The difficulty in inferring abundances of taxa stems largely from the numerous biases incurred through commonly used PCR approaches. A primary reason for this difficulty is that sequence divergence in priming sites affects priming (and subsequently amplification) efficiency directly^12^. Furthermore, there are other factors inherent to the targeted sequence that can bias amplification as well. For example, short sequences are amplified preferentially in amplicon mixes of variable length (e.g. ribosomal DNA), and templates of very low or very high GC content amplify less well. Mitochondrial genes are known to integrate into the nuclear genome as nonfunctional pseudogenes. These often coamplify during PCR^13^, which could also complicate abundance inferences. Another confounding factor in the recovery of abundance estimates from PCR approaches is copy number variation (CNV) of the target locus between taxa^14^. All these factors can lead to flawed abundance estimates from amplicon sequencing data, even with highly conserved priming sites^15,16^.

The many avenues through which biases can be introduced imply that only presence and absence of taxa can be scored reliably from community amplicon sequencing. But as most measures of alpha and beta diversity are dependent on the reliable recovery of taxon abundances^17^, the utility of metabarcoding for diversity assessments has been questioned. Consequently, several suggestions have been made to improve metagenomic assessments of diversity and make abundance estimates possible^18,19^. A short stretch of the mitochondrial Cytochrome Oxidase Subunit I (COI) gene is commonly used as a barcoding marker in animals^20^. While the high variability of COI makes it an ideal choice to identify species or even intraspecific variation, this variation will also enhance priming bias. Hence, other markers with more conserved priming sites have been suggested as potential substitutes for COI^21–24^. Such novel markers, however, can provide less taxonomic resolution^25^ and do not have well-developed sequence reference databases^26^. Another solution is the use of degenerate COI primers, which mitigate PCR bias and allow amplification across a broader taxonomic range, or the design of taxon specific primers^27^. However, factors such as GC content and amplicon length variation will affect amplification irrespective of primer sequence conservation. Thus, alternative approaches suggested to mitigate PCR bias include the increase of DNA template concentrations or reduction of cycle numbers during PCR^28^. As PCR exponentially amplifies DNA templates, amplification bias should significantly increase with the number of PCR cycles. Reducing the number of PCR cycles should mitigate bias and allow for a more accurate correlation of input DNA to recovered reads^16^. PCR-free approaches have also been suggested to exclude amplification bias^29^. The direct sequencing of genomic DNA or sequence capture of barcodes does not require a PCR amplification stage and is hence assumed to provide more accurate predictions of abundance^30–32^. However, such PCR-free methods come with a considerable increase in workload and processing cost (for enrichment, library preparation, and required sequencing coverage), and while they mitigate amplification bias, they are also sensitive to CNV in the target loci.

Despite the evidence for strong PCR biases outlined above, we can capitalize on known elements of PCR predictability and accuracy, such as those shown through applications of quantitative PCR^33^. For example, the proportion of input DNA of a taxon in a community should be tightly correlated to the proportion of recovered reads of that taxon, and amplification bias or CNVs should only affect the slope of this correlation. Recent research has shown that read abundance correction could help in the prediction of species abundances from sequencing data^14,18,34,35^. Since PCR bias is partly induced by sequence composition, it should be similar in closely related taxonomic groups, as has been shown in bacteria^14,36^. Hence, similar correction factors could possibly be derived for closely related taxa, allowing for community level abundance estimates without the need to calibrate a correction model for every taxon in the community.

Considering the afore-mentioned issues, the current study examines the hypotheses that PCR bias in amplicon based metabarcoding can be countered by: (**1**) Choosing appropriate barcode markers with high sequence conservation and/or high levels of primer degeneracy, (**2**) reducing the PCR cycle number and increasing the template concentration during library preparation, (**3**) completely avoiding locus specific amplification and (**4**) identifying and correcting for taxon-specific read abundance bias.

To test these hypotheses, we performed three experiments using DNA and tissue mock communities of taxonomically diverse sets of Hawaiian and Californian arthropods. (**1**) Using eight primer pairs, we test for the effect of different factors on amplification bias as well as qualitative and quantitative community characterization. The targeted amplicons showed varying degrees of sequence conservation. In addition, we used primers of varying degrees of degeneracy. (**2**) In a second experiment, we explored the effect of varying PCR cycle numbers and increasing DNA template concentration during library preparation. (**3**) Finally, we compared the quantitative recovery of taxa by amplicon sequencing with that from metagenomic sequencing of genomic DNA, i.e., completely avoiding amplification with locus specific primers.

## Methods

### Sample collection, mock community preparation

Arthropod samples were collected using beat sheets in native rainforests on the Hawaiian Islands of Maui and Hawaii and oak woodland near the University of California Berkeley campus in the Spring of 2015 and 2016. Specimens were stored in 99-% ethanol, and morphologically identified to order or species where possible (or morphotype when identity was uncertain). We extracted DNA from 43 taxa, representing 19 orders (in the Arachnida, Crustacea, Hexapoda & Myriapoda). DNA extractions were performed on whole bodies using the Qiagen Puregen Kit according to the manufacturer’s protocol (Qiagen, Hilden, Germany). The concentration of each extraction was determined using a Qubit Fluorometer (Thermo Scientific, Waltham, USA) and each sample diluted to a final concentration of 15 ng/µl. We prepared 23 mock communities by pooling randomized volumes of each of the 43 samples. Each pool contained all samples in randomized volumes from 0.7 to 5 µl per sample in increments of 0.1 µl.

### The effect of primer choice on amplification bias

We chose 8 primer combinations amplifying three mitochondrial and four nuclear markers (see Table 1). We had previously generated reference sequences for the specimens in the mock communities for these markers. The primers showed varying degrees of degeneracy and amplified sequences of varying degrees of conservation, from the highly conserved nuclear ribosomal DNA to more variable mitochondrial markers (See Table 2). All primer pairs amplified sequences shorter than 500 bp to achieve an overlap between paired 300 bp Illumina MiSeq reads.

**Table 1:** Targeted genes, primer combinations and primer sequences used in this study, including the average amplicon length (in bp after primer trimming).

Gene	Forward	Sequence 5′-3′	Reverse	Sequence 5′-3′	bp
COI_A	ArF1^5^	GCNCCWGAYATRGCNTTYCCNCG	Fol-degen-rev^27^	TANACYTCNGGRTGNCCRAARAAYCA	418
COI_B	mlCOIintF^53^	GGWACWGGWTGAACWGTWTAYCCYCC	Fol-degen-rev^27^	TANACYTCNGGRTGNCCRAARAAYCA	313
CytB	CB3^54^	GAGGAGCAACTGTAATTACTAA	CB4^54^	AAAAGAAARTATCATTCAGGTTGAAT	358
12SrDNA	12sai^55^	AAACTAGGATTAGATACCCTATTAT	12sbi^55^	AAGAGCGACGGGCGATGTGT	348
18SrDNAV1-2	SSU_FO4^56^	GCTTGTCTCAAAGATTAAGCC	SSU_R22^56^	GCCTGCTGCCTTCCTTGGA	380
18SrDNAV6-7	18s_2F^57^	AACTTAAAGRAATTGACGGA	18s_4R^57^	CKRAGGGCATYACWGACCTGTTAT	304
28SrDNAD6	28s_3F^57^	TTTTGGTAAGCAGAACTGGYG	28s_4R^57^	ABTYGCTACTRCCACYRAGATC	318
Histone H3	H3aF^58^	ATGGCTCGTACCAAGCAGACVGC	H3aR^58^	ATATCCTTRGGCATRATRGTGAC	328

PCRs were run in 10 µl volumes using the Qiagen Multiplex PCR kit, with 1 µl (15 ng) of DNA and 0.5 µl of each 10 µM primer. An optimal annealing temperature of 55 °C for the nuclear and 46 °C for the mitochondrial markers was identified by running gradient PCRs. PCR amplification was performed in two rounds. The first round consisted of 32 cycles using tailed primers, whereas a second indexing PCR was performed on these tails with 6 cycles, to introduce Illumina TruSeq adapters and dual indices. The basic library preparation followed that described in Lange *et al*.^37^. We amplified the mock communities for each of the 8 markers. After each round of PCR, the remaining primer sequences were cleaned from the product with 1X AMpure XP Beads (Beckman Coulter, Indianapolis, USA). The final libraries were quantified with a Qubit Fluorometer, then all samples pooled in equimolar amounts.

### The effect of PCR cycle reduction and DNA template increase on amplification bias

Additionally, we ran a series of PCRs with varying first round cycle numbers. All DNA mock communities were used for this experiment. 4 µl of template DNA (60 ng) were used in a 10 µl PCRs to allow an initial priming of as many template molecules as possible with few PCR rounds. Experiments with 4, 8, 16 and 32 first-round PCR cycles using the primer combination ArF1/Fol-degen-rev were run, followed by second-round indexing PCRs of 26, 22, 14 and 6 cycles. Assuming that primarily locus specific PCR priming bias leads to inaccurate species abundances in community samples, a low number of first-round PCR cycles should reduce this bias. As the indexing PCR is based on the same priming sites (5′-tails introduced in the first round PCR) on all samples, second round priming bias should be of minor concern (See Suppl. Figure 1 for concept visualization). The previously conducted experiment using the primer combination ArF1/Fol-degen-rev with only 1 µl of template DNA (see previous paragraph “*The effect of primer choice on amplification bias*”) was run with the same cycle number as the 32-cycle experiment. This allowed us also to compare the effect of template concentration on amplification bias.

### Metagenomic gDNA sequencing

In addition, we sequenced one of our mock community pools as a metagenomic library. The library was prepared from untreated gDNA using the Illumina TruSeq kit and only six cycles of indexing PCR. We completely avoided amplification with locus specific primers for the metagenomic library preparation. The six-cycle indexing PCR however, was the same for metagenomic and amplicon libraries. This allowed us to estimate the effect of locus specific primer sequences on recovery of different taxa in the communities. Also, the metagenomic data allowed us to estimate the effect of PCR cycle number. With strong PCR amplification bias, the metagenomic pool would be expected to yield significantly more even sequence recovery across taxa, than PCR based libraries.

### Tissue mock communities

To test the applicability of our approach under real conditions, we used mock communities from tissue pools of different Hawaiian taxa. Specimens were identified to species (or morphotype) as described above and defined amounts of tissue of approximately 20 taxa were combined into 30 mock communities. Due to the limited number of samples, we were not able to make exact replicates for the same species for some taxa, but had to make pools with more distant relatives. However, every taxon was represented in multiple pools, so we could correlate biomass with read count. Specimens were dried for 1 hour on Kimwipes at room temperature. Depending on specimen size, specimens were either added whole or cut into sections using a scalpel blade. Each tissue piece was weighed on a micro balance (Mettler-Toledo, Oakland, CA, USA). The respective body parts for each specimen and pool were noted. The final communities contained 5.25–24.12 mg (mean = 15.36 mg) of tissue. They were combined in 2 ml Eppendorf tubes, with a 5 mm stainless steel bead and disrupted by shaking for 2 min at 1,200 hz on a Genogrinder 2010 (OPS Diagnostics, Metuchen, NJ, USA). DNA was extracted from the lysate and the DNA quantified as described above. Mitochondrial COI was amplified from each sample using the primer pairs *mlCOIintF/Fol-degen-rev*.

### Sequencing and sequence analysis

The final pools were sequenced on an Illumina MiSeq, using V3 chemistry and 2 × 300 bp reads according to the manufacturer’s protocol (Illumina, San Diego, USA). Reads were assembled using PEAR^38^ with a minimum overlap of 50 and a minimum quality of 30. The assemblies were quality filtered using the FastX Toolkit^39^ with a minimum of 90-% of bases ≥ Q30. Separate primer pair samples were demultiplexed by marker, using the forward and reverse primer sequences as indices with the *grep* command in UNIX and primer sequences then trimmed using the UNIX *stream EDitor*. We used *grep* to filter all sequences, starting with the forward primer and ending with the reverse primer sequence. Only samples with more than 1,000 reads after quality filtering, assembly, and demultiplexing were retained in the following analyses. Each of the previously generated alignments of reference specimens per marker was used to calculate average uncorrected pairwise genetic distances between all taxa in the reference library (as a measure of conservation of the amplicon) and to create BLAST databases. Using BLASTn against these databases, we quantified the abundance of reads for each of our target taxa and genes in the DNA mock communities. Only the best BLAST hit was retained per sequence. We did not generate separate reference sequences for the tissue mock communities. Instead, an OTU clustering of all concatenated COI sequences from the tissue pools was performed using USEARCH^40^ with a minimum similarity of 97%. The taxonomy of the resulting OTU centroid sequences was assigned using BLAST. Taxon recovery and read abundance to input tissue proportions were analyzed as described above for the DNA pools. Reads of the metagenomic library were blasted against the previously generated reference libraries for all 8 PCR amplicons, to estimate abundances of sequences for the according genes and taxa.

### Qualitative and quantitative community analyses

Using linear regression of the proportion of reads per specimen against its actual proportion in each mock community, we obtained the coefficient of determination (*R* ^2^) and the slope of the associated regression line for each specimen and marker. We used *R* ^2^ as a measure of predictability of the amount of input abundance per taxon vs. the proportion of reads recovered. The slope, on the other hand, served as a measure of fold change between the input proportion of DNA in the mock community and the resulting number of reads. Specifically, we compared slopes to the 1:1 line (representing ideal prediction of recovered reads from input DNA) by taking the difference between the absolute value of the observed slope and 1. A slope of 1, i.e. a perfect association of read count template amount, would translate into a fold change of 0, a slope of 2 into a fold change of +1 and a slope of 0.5 into a fold change of −1. As we did not generate replicates of the gDNA library, we could not perform linear regression for this sample. Instead, a fold change was calculated between the proportion of input DNA for each taxon and the recovered sequences for all eight markers. This fold change was compared to a fold change for amplicon samples of the same genes and taxa.

We then compared alpha diversities between all actual specimen based mock communities and the composition of the same community inferred by sequencing. Alpha diversity (Simpson index & species richness) was calculated using the Vegan package^41^ in R^42^. We also estimated beta diversity between specimen-based and sequence-inferred communities using the Ecodist R package^43^. A low beta diversity indicated an accurate quantitative recovery of the whole community by sequencing. We calculated Jaccard distances as a predictor for qualitative similarity between specimen-based and sequence-inferred communities and Bray Curtis dissimilarities as a measure of quantitative similarity. Alpha and beta diversity were also calculated for the gDNA library. Replicates were generated by randomly resampling the OTU table to a depth of 1,000, 500 times.

We tested for an effect of our different experimental conditions on the above variables, i.e. primer degeneracy (defined as proportion of degenerate bases), amplicon sequence conservation, PCR cycle number and DNA template concentration. Amplicon sequence conservation and primer degeneracy were strongly associated such that high primer degeneracy or high priming site conservation in a targeted amplicon can interchangeably reduce amplification bias in PCR.

### Correcting abundance estimates

We derived correction factors to estimate the relative abundance of taxa. We used the DNA based mock communities for this experiment. Out of 16 total mock communities, we randomly chose 5 and 10 and fitted a regression line for the correlation of input DNA and recovered reads for each taxon in the community. The recovered slope of the regression was used to correct the estimated abundance of the respective taxon for the remaining six community samples. This was done by dividing the recovered proportion of reads per taxon by their corresponding taxon-specific slopes.

## Results

Sequences for most samples were of high quality and coverage. After quality filtering and separation of sequences by loci, we recovered 8,889 ± 4,928 reads per DNA mock community and 14,973 ± 2,268 reads per tissue mock community on average. 2 of the 30 tissue community samples and 6 of the 220 DNA pools had to be excluded due to too low coverage (<1000 reads). The metagenomic library yielded 835.87 × 10^6^ bp in 3.85 × 10^6^ sequences.

**Table 2:** Summary of amplified genes and experimental conditions as well as result from our amplicon sequencing experiments. The sample size (N) of each experiment is shown. The analyzed variables include: amount of DNA per PCR (ng DNA), PCR cycle number (Cycle #), primer degeneracy (Degen.) and average pairwise distance (Dist.) of markers in our mock communities and the average length and standard deviation of each amplicon after primer removal (bp).The table also shows the mean and standard deviation of the coefficient of determination (R^2^) of the association between input DNA and recovered read count, the fold change (FC) between DNA and recovered reads, Simpson indexes (α) species richness (SR), as well as Bray Curtis dissimilarity (β_BC_) and Jaccard distances (β_JC_) between specimen based and sequence based communities.

Gene	Primer	N	ng DNA	Cycle #	Degen.	Dist.	bp	R^2^	FC	α	SR	β_BC_	β_JC_
COI_A	ArF1/Fol-degen-rev	23	60	4	0.21	0.26	418 ± 0.38	0.74 ± 0.17	−1.63 ± 4.25	0.93 ± 0.03	40.78 ± 0.55	0.43 ± 0.03	0.05 ± 0.01
COI_A	ArF1/Fol-degen-rev	23	60	8	0.21	0.26	418 ± 0.38	0.67 ± 0.11	−1.75 ± 2.62	0.93 ± 0.02	41.00 ± 0.00	0.43 ± 0.03	0.05 ± 0.00
COI_A	ArF1/Fol-degen-rev	23	60	16	0.21	0.26	418 ± 0.38	0.87 ± 0.08	−1.07 ± 2.97	0.93 ± 0.02	41.00 ± 0.00	0.41 ± 0.02	0.05 ± 0.00
COI_A	ArF1/Fol-degen-rev	23	60	32	0.21	0.26	418 ± 0.38	0.86 ± 0.08	−0.87 ± 2.00	0.95 ± 0.01	41.00 ± 0.21	0.38 ± 0.02	0.05 ± 0.01
COI_A	ArF1/Fol-degen-rev	16	15	32	0.21	0.26	418 ± 0.38	0.88 ± 0.08	−1.07 ± 2.38	0.95 ± 0.01	40.96 ± 0.00	0.39 ± 0.02	0.05 ± 0.00
COI_B	mlCOIintF/Fol-degen-rev	16	15	32	0.17	0.25	313 ± 0.38	0.88 ± 0.11	−1.34 ± 3.047	0.95 ± 0.01	41.38 ± 0.50	0.42 ± 0.02	0.04 ± 0.01
CytB	CB3/CB4	16	15	32	0.01	0.31	328 ± 0.00	0.55 ± 0.23	−11.33 ± 22.50	0.82 ± 0.05	29.69 ± 1.58	0.70 ± 0.03	0.31 ± 0.04
12SrDNA	12sai/12sbi	16	15	32	0.00	0.28	348 ± 11.89	0.64 ± 0.19	−0.60 ± 6.18	0.76 ± 0.05	17.06 ± 1.61	0.78 ± 0.02	0.60 ± 0.04
18SrDNAV1-2	SSU_FO4/SSU_R22	16	15	32	0.00	0.12	380 ± 12.06	0.84 ± 0.16	−1.79 ± 4.97	0.93 ± 0.01	41.69 ± 0.48	0.44 ± 0.02	0.03 ± 0.02
18SrDNAV5-6	18s_2F/18s_4R	16	15	32	0.06	0.09	304 ± 41.39	0.86 ± 0.17	−1.89 ± 6.69	0.93 ± 0.01	40.88 ± 0.34	0.41 ± 0.02	0.05 ± 0.01
28SrDNAD6	28s_3F/28s_4R	16	15	32	0.08	0.16	318 ± 12.20	0.81 ± 0.19	−1.20 ± 3.86	0.92 ± 0.01	36.75 ± 0.58	0.47 ± 0.02	0.15 ± 0.01
H3	H3aF/H3aR	16	15	32	0.05	0.21	328 ± 0.00	0.54 ± 0.27	−4.12 ± 9.97	0.93 ± 0.01	35.81 ± 3.45	0.53 ± 0.03	0.17 ± 0.08

### Qualitative and quantitative community analyses based on DNA pools

**Figure 1:**
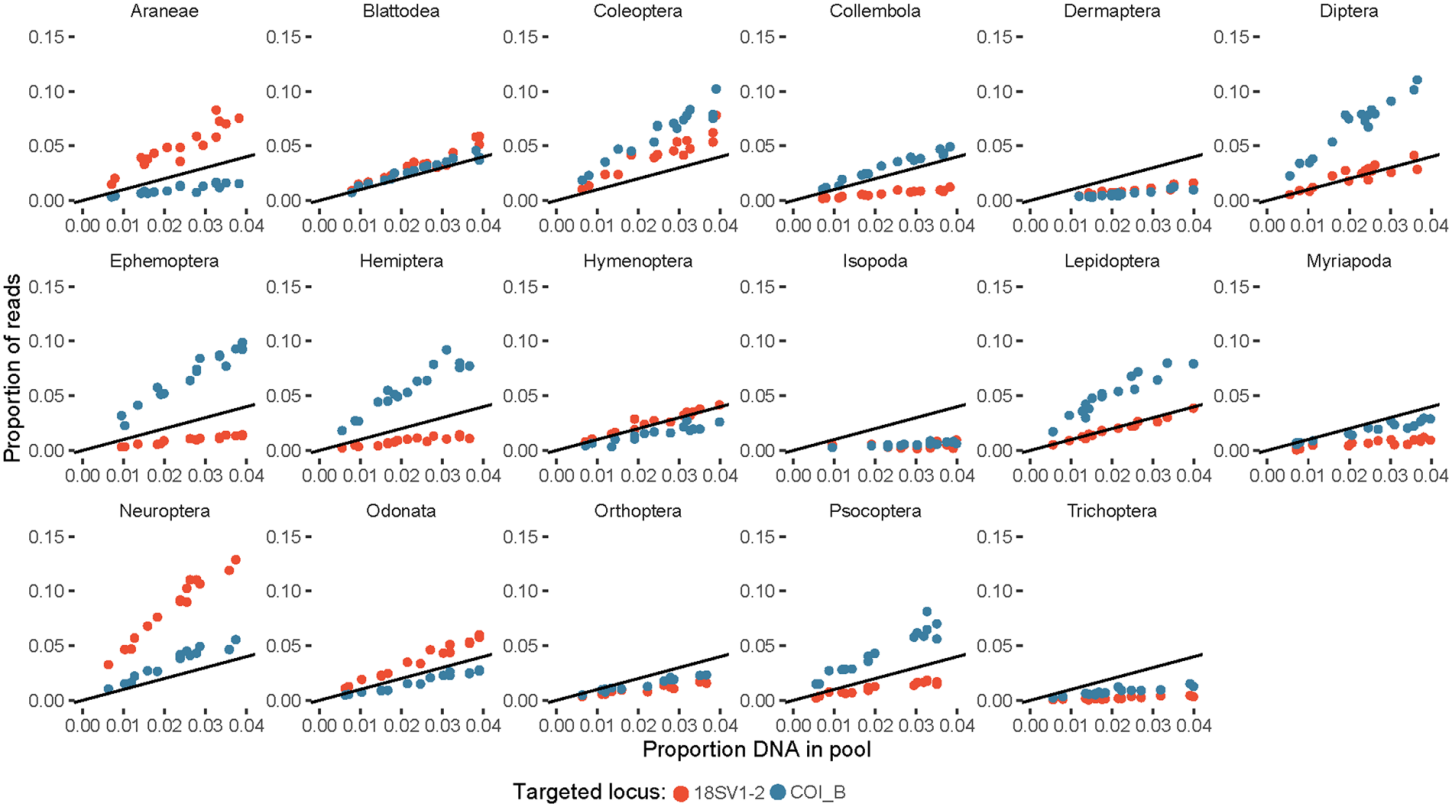
Proportion of input DNA against the proportion of recovered reads per taxon for various arthropod orders. Plots are based on DNA mock communities using mitochondrial COI (blue) and nuclear 18SrDNA (red) markers. 1:1 lines are in black.

In our experiment testing 8 primer pairs, we found a positive linear association and a tight correlation, i.e., a high coefficient of determination (R^2^), between recovered read counts and input DNA for most arthropod taxa (Table 2 and Fig. 1). This association was independent of the amount of the target taxon or other taxa in the mock community. The slope of the association varied across taxa and markers, as evidenced by the highly conserved nuclear ribosomal 18SrDNA, as well as the variable mitochondrial COI (Fig. 1).

**Figure 2:**
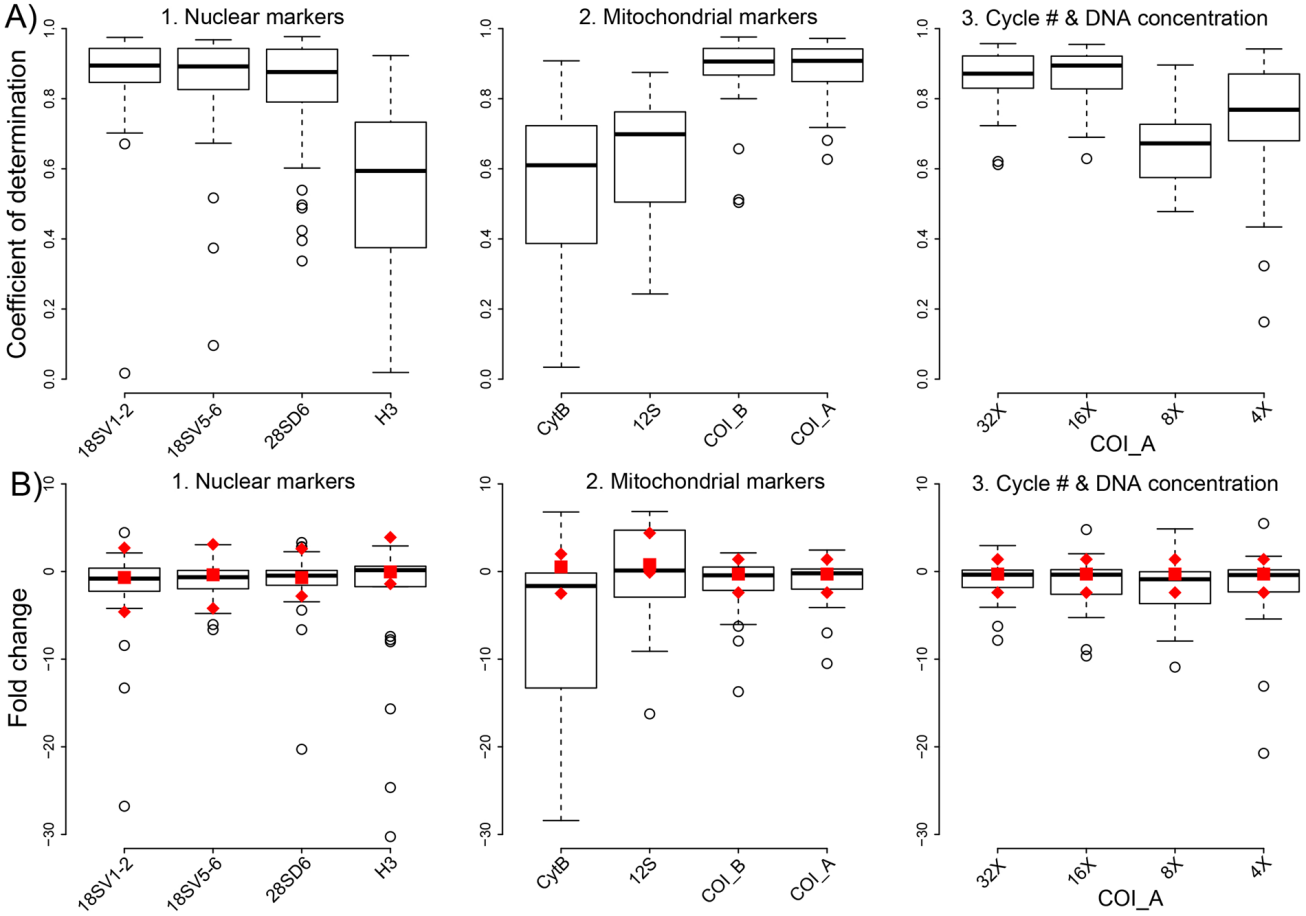
(**A**) Coefficient of determination (R^2^) of the linear association between input DNA and recovered read proportions for 43 arthropod taxa. The boxplots show R^2^ for 1. nuclear and 2. mitochondrial markers, as well as 3. mitochondrial COI amplified with varying first round PCR cycle numbers and increased amount of DNA template during PCR. (**B**) Fold change between input DNA and recovered read proportions for the same taxa and experimental conditions. Red squares indicate the median fold change for the same taxa and loci based on a gDNA library prepared without locus specific amplification. Red diamonds indicate the location of upper and lower whiskers for the boxplots of the same gDNA samples.

The coefficient of determination between input DNA and recovered reads was relatively high for most targeted primer pairs (Table 2, Fig. 2A). In other words., the amount of a taxon in a DNA mock community was usually well correlated to the recovered read count. The fold change between input DNA and recovered reads was mostly narrowly distributed around an actual 1:1 association (Table 2, Fig. 2B). Most markers thus allowed a relatively good prediction of taxon abundances from read counts.

We found a strong association of primer degeneracy and amplicon sequence conservation with the coefficient of determination, as well as fold change. A high conservation of the targeted amplicon or high degeneracy of the used primer pair led to a significantly better correlation between input DNA and recovered reads (Figs 2 and 4A) (Pairwise Wilcoxon test, FDR corrected *P* < 0.05). At the same time, the variation of fold change was significantly reduced by sequence conservation and primer degeneracy (Levene’s test, *P* < 0.05) (Table 2, Figs 2 and 4B). The lowest R^2^ and highest variation for fold change (i.e. worst predictability of taxon abundance from read count) was consistently found for 12SrDNA, CytochromeB and H3, which all showed a fairly high amount of sequence variation coupled with little primer degeneracy. While the two-targeted COI amplicons also had a relatively high amount of amplicon sequence variation, the primers used here were highly degenerate. The nuclear ribosomal markers, in contrast, were highly conserved.

Our experiment of varying PCR cycle numbers and increasing DNA template concentration did not reveal any effect of DNA template concentration on either R^2^ or fold change. Fold change was also unaffected by first round PCR cycle numbers. Contrary to our expectations, R^2^ showed a significant drop below 16 PCR cycles (Pairwise Wilcoxon test, FDR corrected *P* < 0.05) (Fig. 2A and B, Table 2). The association of input DNA and recovered reads was thus less predictable at low PCR cycle numbers.

The fold change between input DNA and recovered reads was very similar between amplicon libraries and our PCR free gDNA library. However, the variation of fold change was lower for the gDNA libraries (Fig. 2B, Suppl. Table 1). A major difference was found for those markers in the amplicon libraries which showed the highest sequence variation (i.e., 12SrDNA, CytochromeB & H3) (Pairwise Wilcoxon test, FDR corrected *P* < 0.05). In the gDNA libraries, the variation of fold change for these loci was considerably reduced and well comparable to the other loci (Fig. 2B).

**Figure 3:**
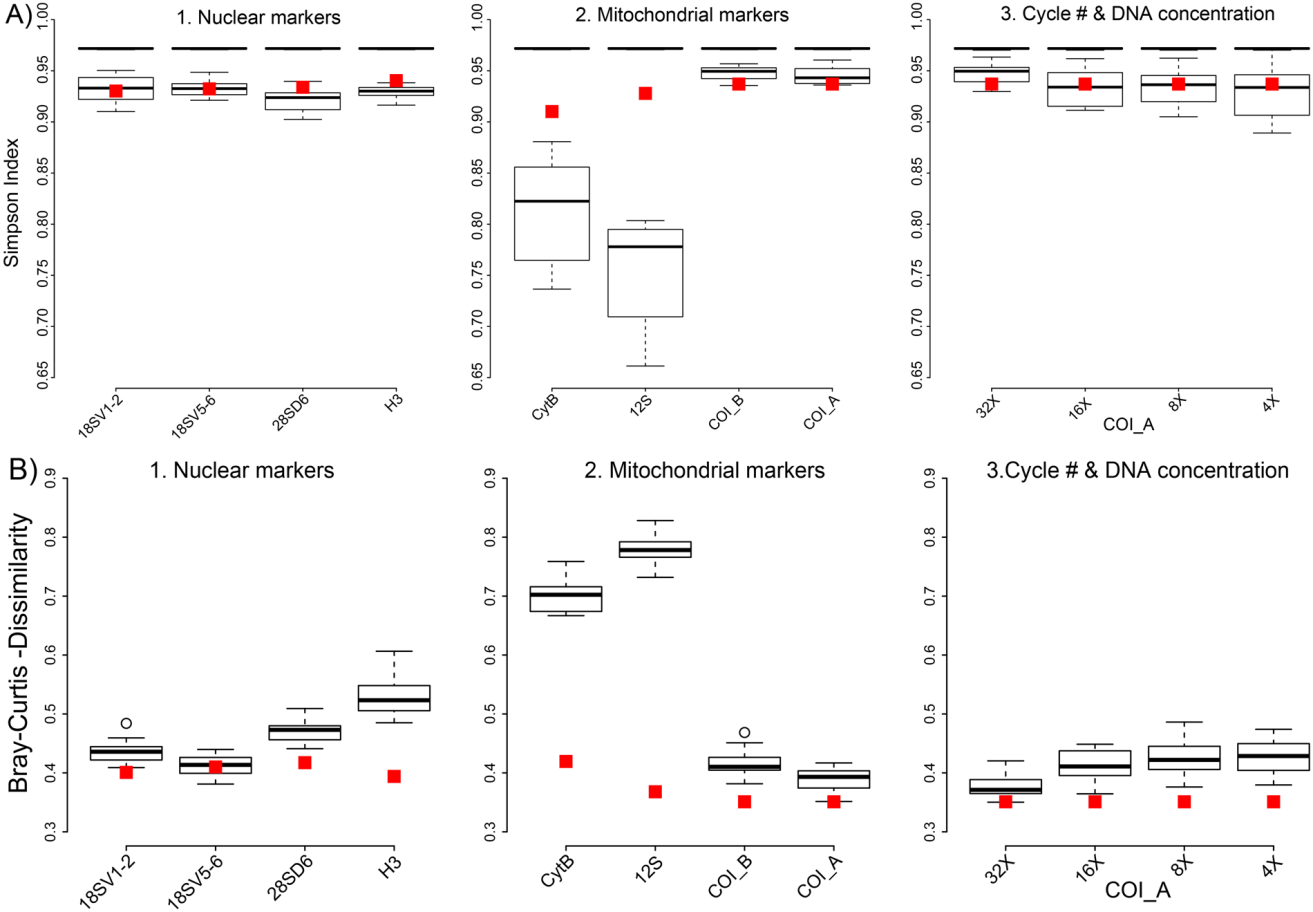
(**A**) Alpha diversity (Simpson Index) of arthropod mock communities. The upper black bar shows the median alpha diversity of the actual communities based on morphospecies assignments. The boxplots show alpha diversity for the same communities based on DNA sequencing for 1. nuclear and 2. mitochondrial markers, and 3. for mitochondrial COI at varying PCR cycle numbers and increased DNA template amount during PCR. Red squares indicate alpha diversity for the same loci based on a genomic DNA sample prepared without locus specific amplification. (**B**) Beta diversity (Bray Curtis dissimilarity) between actual morphospecies based mock communities and sequence based analyses. The boxplots and red present the same experimental conditions as described above. Red squares indicate beta diversity for the same loci and based on a genomic DNA sample prepared without locus specific amplification.

**Figure 4:**
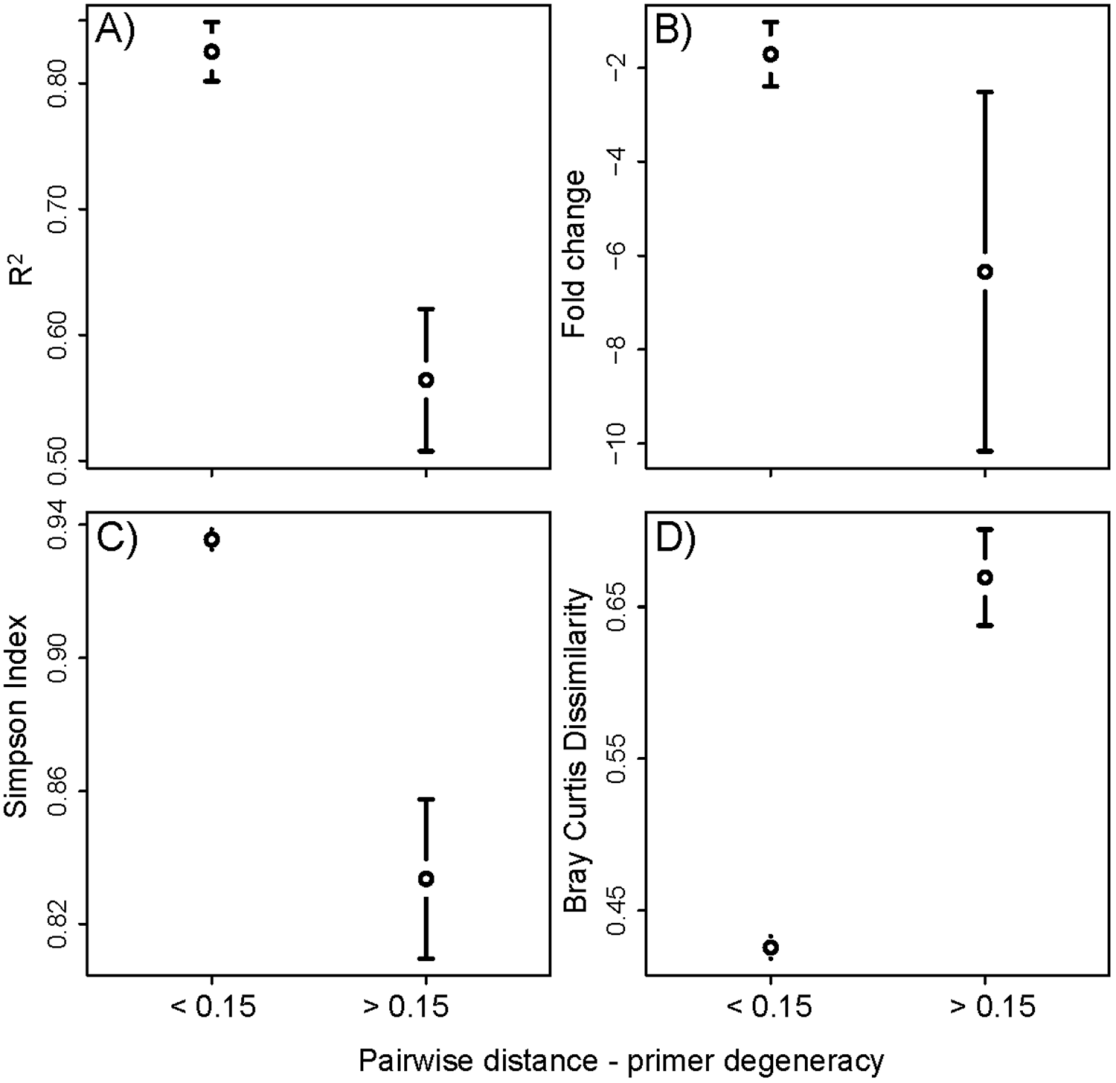
(**A**) Coefficient of determination (R^2^) of the linear association between input DNA and recovered read abundance for two marker groups and 43 arthropod taxa. The groups are based on the difference between the average pairwise genetic distance of taxa for the according marker and the degeneracy of the primer pair used to amplify the locus. Group one comprises amplicons with a high sequence conservation and/or a high primer degeneracy. Group two comprises variable amplicons, with little primer degeneracy. (**B**) Fold change between input DNA and recovered reads for the same taxa and markers. (**C**) Alpha diversity (Simpson index) for the same marker groups. (**D**) Beta diversity (Bray Curtis dissimilarity) between specimen based and sequence based communities for the same marker groups. The plots show the mean and the 95% confidence interval.

Similar to R^2^ and fold change, the alpha diversity of our DNA mock communities in our 8-primer experiment was also strongly associated with primer degeneracy and amplicon sequence conservation (Table 2, Figs 3A and 4C, Suppl. Figure 2A). Significantly increased Simpson indexes and species richness were found for loci with high sequence conservation or highly degenerate primers (Pairwise Wilcoxon test, FDR corrected *P* < 0.05). A similar association was found for beta diversity. Jaccard distance and Bray Curtis dissimilarity between the actual specimen-based mock community and the same communities inferred from sequence analysis, decreased significantly with amplicon conservation and primer degeneracy (Pairwise Wilcoxon test, FDR corrected *P* < 0.05) (Figs 3B and 4D, Suppl. Figure 2B).

Our experiment on PCR cycle number and template concentration revealed a slight, but significant decrease of alpha diversity and increase of beta diversity for a decrease of PCR cycle numbers (Pairwise Wilcoxon test, FDR corrected *P* < 0.05). At low PCR cycle numbers, the community composition inferred from sequencing was thus more different from the actual community. In contrast, DNA template concentration did not have a significant effect (Table 2, Fig. 3A and B).

The average recovered species richness and Simpson indexes for our amplicon sequencing samples was significantly lower than the richness of the actual mock community (Pairwise Wilcoxon test, FDR corrected *P < *0.05). In other words, not all taxa present in our mock communities were recovered by sequencing. However, the difference was small for most loci (Table 2, Fig. 3A, Suppl. Figure 2A). We found a pronounced difference between qualitative and quantitative estimates of beta diversity. The Jaccard distances between specimen-based mock communities and the same communities derived from sequencing were very low for most amplicons (Suppl. Figure 2B). Bray Curtis dissimilarity, which incorporates taxon abundances, was significantly higher for all loci (Table 2, Fig. 3B) (Pairwise Wilcoxon test, FDR corrected *P* < 0.05).

The gDNA based library generally showed slightly lower Bray Curtis dissimilarities and higher Simpson indexes than the amplicon libraries. However, a pronounced effect was only found for amplicons with high sequence variation and low primer degeneracy (12SrDNA, CytochromeB & H3) (Fig. 3A and B, Suppl. Table 1).

In summary, targeting highly conserved loci, or using highly degenerate primers, led to a considerable improvement of the association of input DNA and recovered read count and more reliable qualitative and quantitative recovery of species diversity from communities (Fig. 4).

### Tissue mock communities

**Figure 5:**
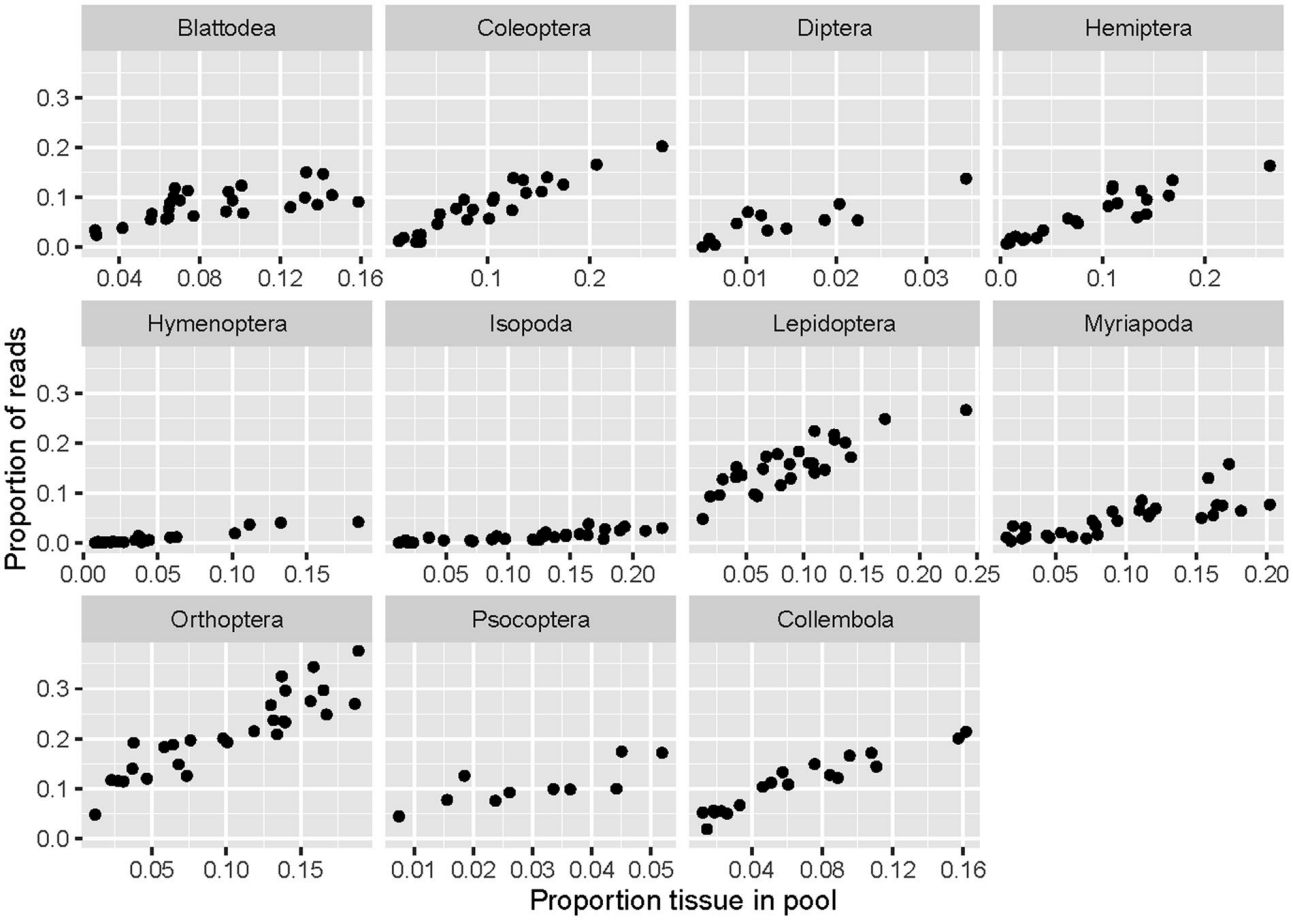
Exemplary associations of proportion of tissue and proportion of recovered reads for different arthropod orders. The plots are based on tissue mock communities amplified using COI_B (Table 2).

We found no significant difference in fold-change between DNA-based and tissue-based mock communities (Suppl. Figure 3B). However, tissue pools showed a lower coefficient of determination per taxon, than DNA pools (Suppl. Figure 3A) (Mann Whitney test, *P* < 0.001), i.e., the association between input tissue and recovered reads was not as predictable. Nevertheless, the amount of tissue per taxon was still well correlated with the read count (Fig. 5). Replicates of the same taxon (Collembola, Isopoda & Myriapoda) from DNA and tissue pools, recovered very similar associations between input tissue/DNA and recovered reads (Fold change read count vs. input tissue|input DNA: FC_Collembola_ = 0.152|0.158; FC_Isopoda_ = −6.576|−4.917; FC_Myriapoda_ = −0.965|−0.490; Suppl. Figure 4). The Simpson indexes of the actual tissue based communities were significantly correlated to those derived from sequencing (R^2^ = 0.532) (Suppl. Figure 3C). Moreover, the recovered Bray Curtis dissimilarities between specimen and sequence based communities were not higher than those found for our DNA pools (Suppl. Figure 3D).

### Correcting abundance estimates

**Figure 6:**
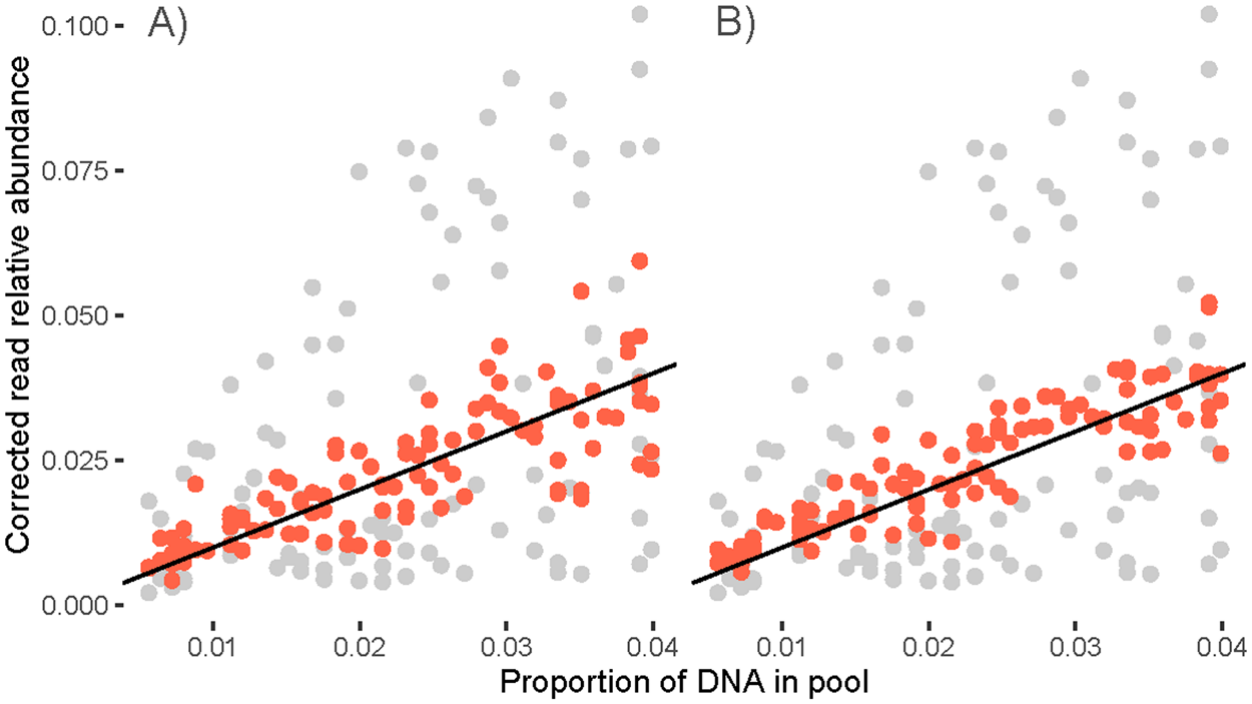
Effect of correcting read abundances on quantitative taxon recovery. Uncorrected association of actual abundance and recovered read proportion for 43 arthropod taxa (grey dots) and after applying the taxon specific slope of the association between input DNA and read count as correction factor for the read abundance (red dots) using (**A**) 5 mock communities or (**B**) 10 mock communities to derive the correction factors. The black lines represent the 1:1 lines.

Each taxon showed a predictable fold change between the proportion of input DNA and recovered reads. But, due to taxon-specific slopes, a simple association of the proportion of input DNA and recovered reads for all taxa in six mock communities suggests no correlation (R^2^ = 0.09; *P* > 0.05; Fig. 6). By using 5 mock communities to derive taxon-specific correction factors, a significant correlation was found (R^2^ = 0.59; *P* < 0.05; Fig. 6A). This correlation improved when 10 mock communities were used to derive corrections factors (R^2^ = 0.82, *P* < 0.05; Fig. 6B). The amount of input DNA could thus be fairly accurately predicted from mock communities for most taxa. Read abundance correction also led to significantly decreased Bray Curtis dissimilarities between specimen based and sequence based communities (Pairwise Wilcoxon test, FDR corrected *P* < 0.05) (Supplementary Figure 5).

## Discussion

### Mitigating amplification bias in metabarcoding

We found that metabarcoding accurately recovers the qualitative species composition of diverse arthropod communities, giving rise to very similar species richness and low Jaccard distances between specimen-based and sequence-based community samples. However, we found pronounced quantitative bias in our sequence based community analyses. This bias can partly be attributed to differential amplification due to priming efficiency during PCR. This amplification bias can be alleviated considerably by using degenerate primers (but note that increasing primer degeneracy also increases undesired amplification) and/or targeting amplicons with high priming site conservation. Yet, even under optimized PCR conditions or completely avoiding locus specific amplification in our metagenomic library, we found considerable differences in read abundances across taxa. These differences are possibly caused by the six cycles of indexing PCR, but considering the fact that indexing PCR primers for all amplicons are targeting exactly the same priming sequence, priming bias alone seems unlikely. Factors inherent to the target sequence could cause amplification bias during indexing PCR, e.g. length variation or GC bias. However, these factors should affect the first round PCR as well and a removal of first round PCR did not yield a strong effect. Copy number variation of the target loci^14^ is another possible reason for read abundance differences. All the amplified loci in our study are present in multiple copies in each cell. Mitochondrial copy number even varies considerably between different organs in a single organism^44^. And different arthropod taxa carry different ratios of tissue types with different mitochondrial content, e.g. muscles in flying and non-flying species.

Interestingly, a reduction of first round PCR cycles below 16 did not improve abundance estimates, and even led to a less predictable association of read count and taxon abundances (i.e. reduced R^2^). This may be due to a higher stochasticity of amplification in the initial rounds of PCR, before the reaction reaches the exponential phase. Our findings are also in line with work from Sipos *et al*.^45^, suggesting only a small effect of PCR cycle number, and a major effect of primer template mismatches, on amplification bias. This finding is encouraging for researchers seeking to characterize historical museum collections. With only small amounts of DNA remaining, such samples have to be processed with high PCR cycle numbers to achieve amplification.

PCR-free analyses have been suggested as possible means for quantitative community analysis. This approach circumvents amplification bias^30^ and has been shown to result in better recovery of taxa from diverse communities^46^. Indeed, even our best primer combinations did not recover all taxa from mock communities, as indicated by consistently lower species richness of sequence based over actual communities. This suggests PCR free approaches as the method of choice for exhaustive community analyses, where the recovery of all taxa is of critical importance. However, quantitative analyses using PCR free methods will be similarly sensitive to CNVs of the target genes. Also, an amplicon sequencing-based approach is much more cost efficient and involves a greatly simplified workflow, making it the method of choice for large-scale community analyses.

### Abundance estimates by metabarcoding

Due to biases in read abundance, metabarcoding does not allow direct estimation of actual species abundances. However, despite the observed taxonomic bias of read abundances, the amount of recovered reads was correlated in a very predictable way with the amount of input DNA. Similar results have been found for microorganisms^47,48^. The correction of read abundances can thus yield an approximation of taxon abundances in a community^18,34^. For a quantitative analysis by metabarcoding, the expected taxa in the studied system and the taxon specific PCR amplification bias need to be known. The identification of correction factors involves considerable effort and is not feasible in unknown ecosystems or for simple exploratory work. But for large scale and long-term studies in one ecosystem, the effort could pay off. As every primer combination results in different fold change for different taxa, it is advisable to focus on only a few or even a single marker for such quantitative optimization. This approach seems particularly suitable for comparative studies on abundance changes of a subset of target taxa such as invasive species across different sites. Both, copy number variation and sequence composition could affect abundance biases between taxa. Both these factors are probably more similar between closely related taxa, i.e., suggesting a similar bias between them^14,36^. Correction factors thus may not have to be developed for every species, but could be derived for groups of higher taxa. We are currently analyzing this possibility in a larger dataset of arthropod taxa (Krehenwinkel *et al*. in prep.).

### Metabarcoding and mitochondrial COI – a perfect match

Even nuclear ribosomal markers with highly conserved priming sites did not yield significantly better qualitative or quantitative results than degenerate COI primers. In contrast to nuclear rDNA, COI is more variable and can distinguish even recently diverged species. While 18SrDNA and 28SrDNA performed well in our analysis, they may be too conserved for many barcoding applications^25,49^. Our study was mostly based on quite divergent taxa, which are still differentiated using conserved markers like nuclear rDNA. However, nuclear rDNA would likely fail to distinguish recently diverged species. Compared to other markers, COI is distinguished by an exceptionally well-developed reference database^26^, which often allows species identification. Recent studies suggested alternative primers to COI^23,24^. Indeed, different markers are advisable for certain taxa; for example, we were unable to amplify some Acari and Hymenoptera with COI. Mitochondrial markers bring along problems such as NUMTS^13^ and their genealogy can be strongly affected by bacterial infections^50^ or paternal gene flow^51^. Hence, a suitable nuclear marker would be recommendable for future studies. The internal transcribed spacers of the ribosomal cluster are promising targets; they have already been successfully applied in fungal taxonomy^52^. But, as more genomic data becomes available, a multitude of novel markers may be discovered in the coming years.

## Conclusion

PCR amplification bias can be significantly mitigated by degenerate primers or by targeting amplicons with conserved priming sites. Apart from PCR bias, copy number variation of the target locus could contribute to read abundance differences between taxa, affecting PCR-free and amplicon-based approaches alike. Taxon-specific correction factors can be applied to derive abundance estimates provided researchers have a solid understanding of the taxonomic composition of the community of interest.

### Data accessibility

Illumina reads and analysis tables are available in the Dryad Digital Repository. doi:10.5061/dryad.fs728

1. Read files for all analyzed sequences
2. Analysis tables containing DNA or tissue proportions and read counts for each taxon and each mock community

## Electronic supplementary material

Supplement
