# Supplementary material for: Estimating and mitigating amplification bias in qualitative and quantitative arthropod metabarcoding
Source: Sci Rep. 2017 Dec 15;7:17668. doi: 10.1038/s41598-017-17333-x (PMC5732254; doi:10.1038/s41598-017-17333-x)
Supplement: Supplementary file 1 — Supplement [file 41598_2017_17333_MOESM1_ESM.pdf]

Supplementary material

Estimating and mitigating amplification bias in qualitative and quantitative arthropod metabarcoding

Henrik Krehenwinkel[1 2], Madeline Wolf[1], Jun Ying Lim[1], Andrew J. Rominger[1], Warren B. Simison[2], Rosemary G. Gillespie[1]

[1]Department of Environmental Sciences, Policy and Management

University of California Berkeley

Mulford Hall, Berkeley, California, USA

[2]Center for Comparative Genomics

California Academy of Sciences

Music Concourse Drive, San Francisco, California, USA

Krehenwinkel@berkeley.edu

+1-510-646-3409

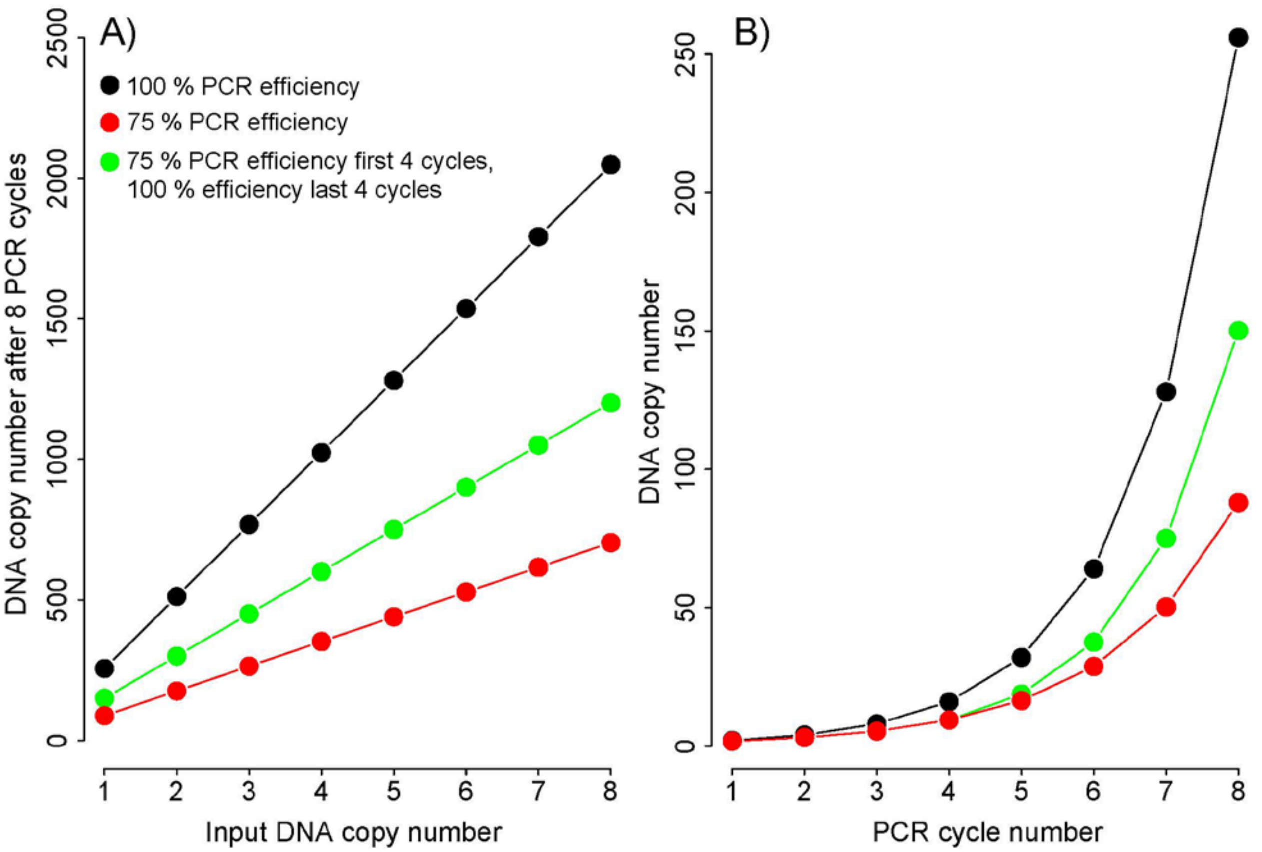

Supplementary Figure 1 A) *Hypothetical association of input DNA copy number and recovered DNA copy number after 8 PCR cycles for two taxa with a difference in PCR efficiency of 25 % (red and black circles). By using a nested PCR on conserved priming sites after 4 cycles, the recovered copy number is corrected (green circles), leading to less biased abundance estimates.* ***B)*** *Association of PCR cycle number and DNA copy number for the same taxa. By removing PCR bias after four cycles, the difference in copy number between the two taxa is reduced.*

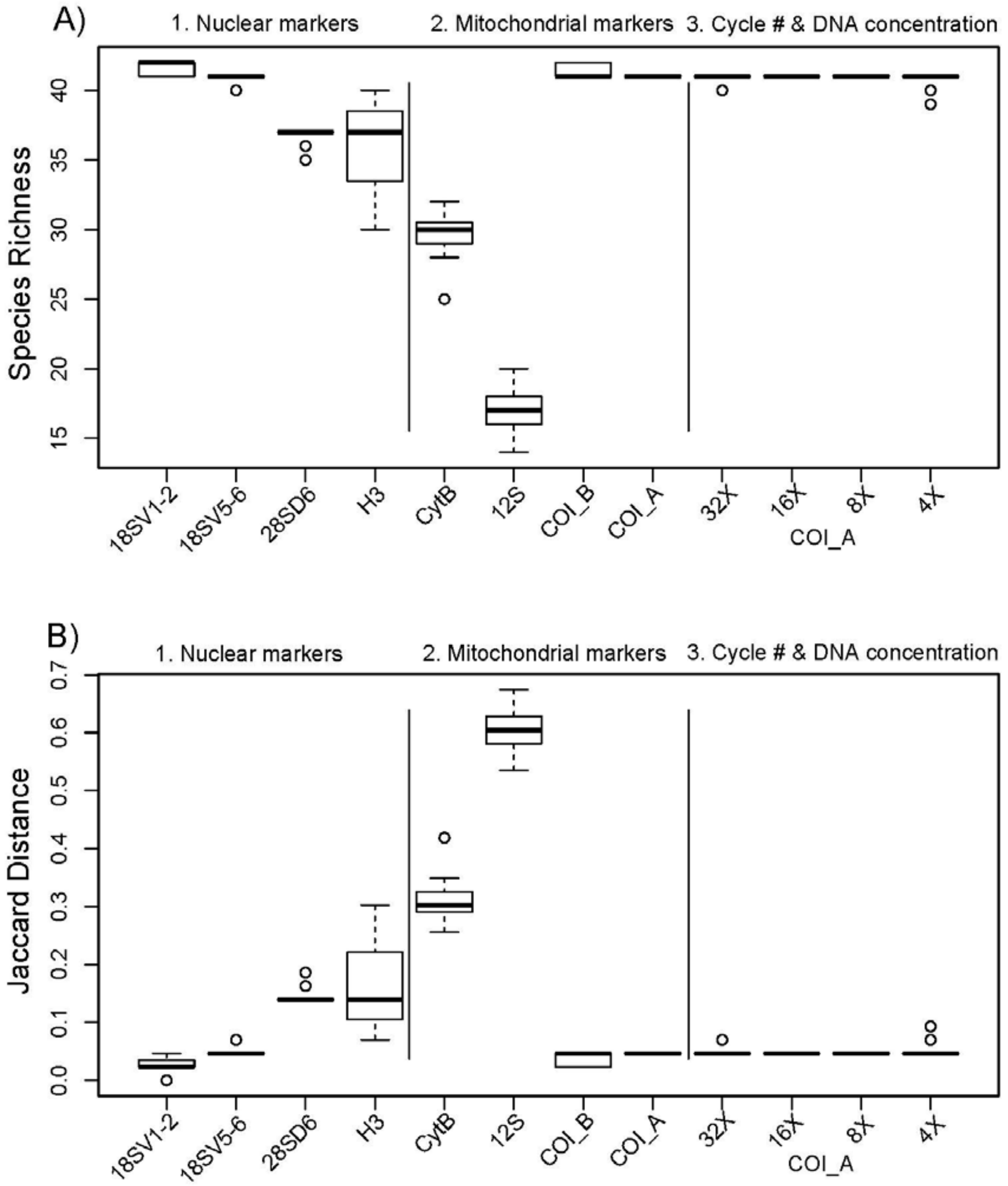

Supplementary Figure 2 **A)** *Species richness for all mock communities of 43 taxa, based on DNA sequencing for* ***1.*** *nuclear and* ***2.*** *mitochondrial markers, and* ***3.*** *for mitochondrial COI at varying PCR cycle numbers and increased DNA template amount during PCR.* ***B)*** *Beta diversity (Jaccard distances) between actual morphospecies based mock communities and sequence based analyses. The boxplots present the same experimental conditions as described above.*

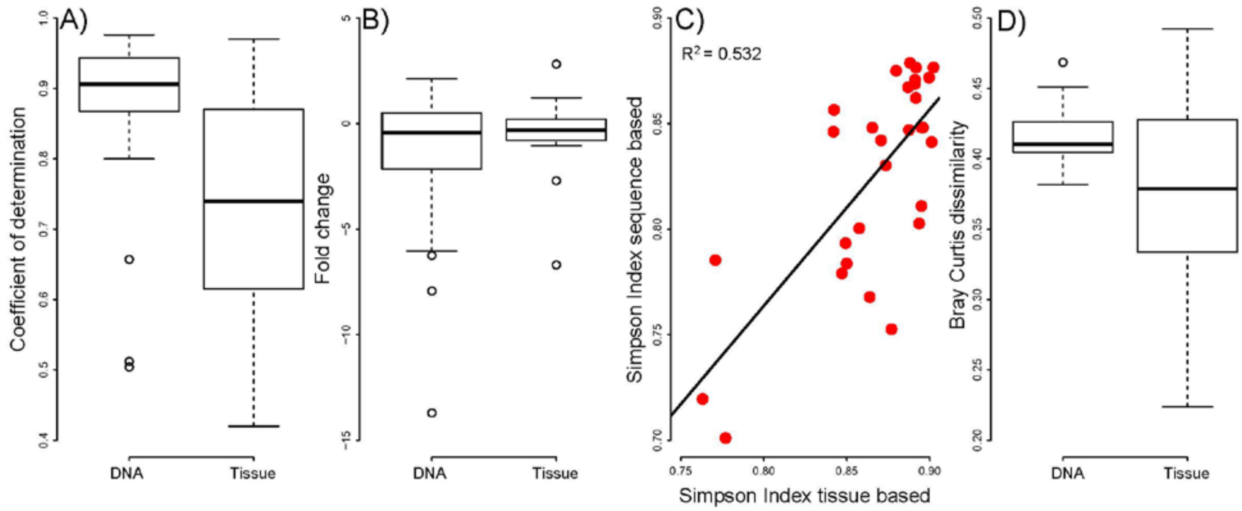

Supplementary Figure 3 A) *Coefficients of determination (R^2) of the linear association between actual abundance and read abundance for DNA based and tissue based mock communities.* ***B)*** *Fold change between actual abundance and read abundance for the same communities.* ***C)*** *Association of alpha diversity (Simpson indexes) based on actual abundances and sequence based abundance estimates for tissue mock communities.* ***D)*** *Beta diversity (Bray Curtis dissimilarity) between actual specimen based and sequence based communities and based on DNA or tissue mock communities.*

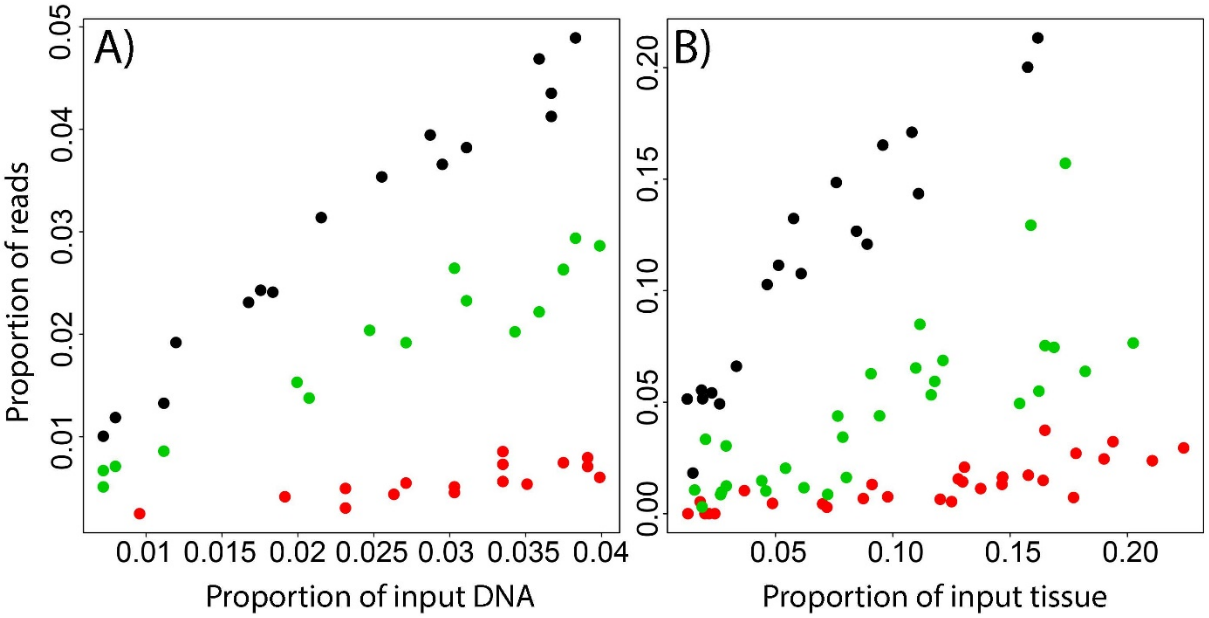

Supplementary Figure 4 A) *Association of the proportion of input DNA and recovered reads for three arthropod species (red:* Porcellio scaber, green: *Proteroiulus fuscus*, black: Tomocerus minor*) and* ***B)*** *for the same three species based on tissue pools.*

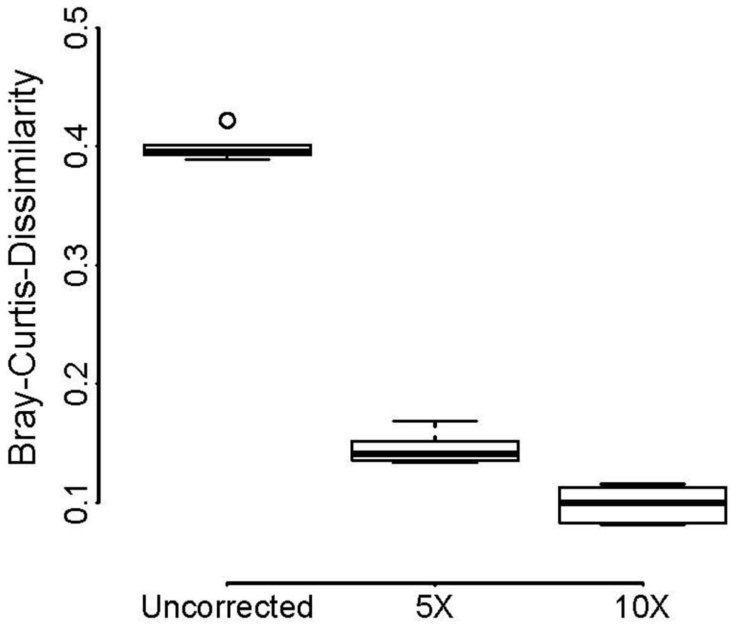

Supplementary Figure 5 *Bray Curtis dissimilarity between specimen based and sequencing based community samples for uncorrected read data, and using five or ten mock communities to derive corrections factors and correct read abundances.*

Supplementary Table 1 *Mean and standard deviation of fold change of input DNA and read abundance, as well as alpha diversity (Simpson index) and beta diversity (Bray Curtis dissimilarity) for PCR amplicon based and metagenomic samples. Fold change was calculated in relation to a hypothetical 1:1 association. The table shows all eight targeted loci. Amplicons were prepared with 32 PCR cycles following six cycles of indexing PCR. Metagenomic samples were prepared from untreated genomic DNA with only six cycles of indexing PCR.*

<table>
<tr><th>Primer pair</th><th>$FC_{amplicon}$</th><th>FC_{gDNA}</th><th>$\alpha_{amplicon}$</th><th>α_{gDNA}</th><th>$B_{amplicon}$</th><th>B_{gDNA}</th></tr>
<tr><td>ArF1/Fol-degen-rev</td><td>-1.073 ± 2.384</td><td rowspan="2">-0.454 ± 2.11</td><td>0.945 ± 0.008</td><td rowspan="2">0.937 ± 0.002</td><td>0.389 ± 0.019</td><td rowspan="2">0.347 ± 0.008</td></tr>
<tr><td>mlCOIintF/Fol-degen-rev</td><td>-1.336 ± 3.047</td><td>0.947 ± 0.007</td><td>0.417 ± 0.021</td></tr>
<tr><td>CB3/CB4</td><td>-11.330 ± 22.497</td><td>0.312 ± 2.069</td><td>0.815 ± 0.050</td><td>0.910 ± 0.004</td><td>0.702 ± 0.028</td><td>0.420 ± 0.009</td></tr>
<tr><td>12sai/12sbi</td><td>-0.603 ± 6.180</td><td>0.691 ± 1.462</td><td>0.756 ± 0.047</td><td>0.928 ± 0.002</td><td>0.778 ± 0.023</td><td>0.369 ± 0.012</td></tr>
<tr><td>SSU_FO4/SSU_R22</td><td>-1.792 ± 4.965</td><td>-0.770 ± 2.491</td><td>0.931 ± 0.013</td><td>0.940 ± 0.001</td><td>0.435 ± 0.019</td><td>0.403 ± 0.005</td></tr>
<tr><td>18s_2F/18s_4R</td><td>-1.894 ± 6.694</td><td>-0.583 ± 2.637</td><td>0.933 ± 0.009</td><td>0.934 ± 0.002</td><td>0.412 ± 0.017</td><td>0.405 ± 0.007</td></tr>
<tr><td>28s_3F/28s_4R</td><td>-1.198 ± 3.857</td><td>-0.644 ± 2.221</td><td>0.922 ± 0.011</td><td>0.930 ± 0.002</td><td>0.470 ± 0.018</td><td>0.418 ± 0.005</td></tr>
<tr><td>H3aF/H3aR</td><td>-4.124 ± 9.968</td><td>0.680 ± 1.715</td><td>0.925 ± 0.006</td><td>0.932 ± 0.002</td><td>0.528 ± 0.031</td><td>0.389 ± 0.013</td></tr>
</table>
